# Supplementary figures and images for: ATRPred: A machine learning based tool for clinical decision making of anti-TNF treatment in rheumatoid arthritis patients
Source: PLoS Comput Biol. 2022 Jul 5;18(7):e1010204. doi: 10.1371/journal.pcbi.1010204 (PMC9321399; doi:10.1371/journal.pcbi.1010204)

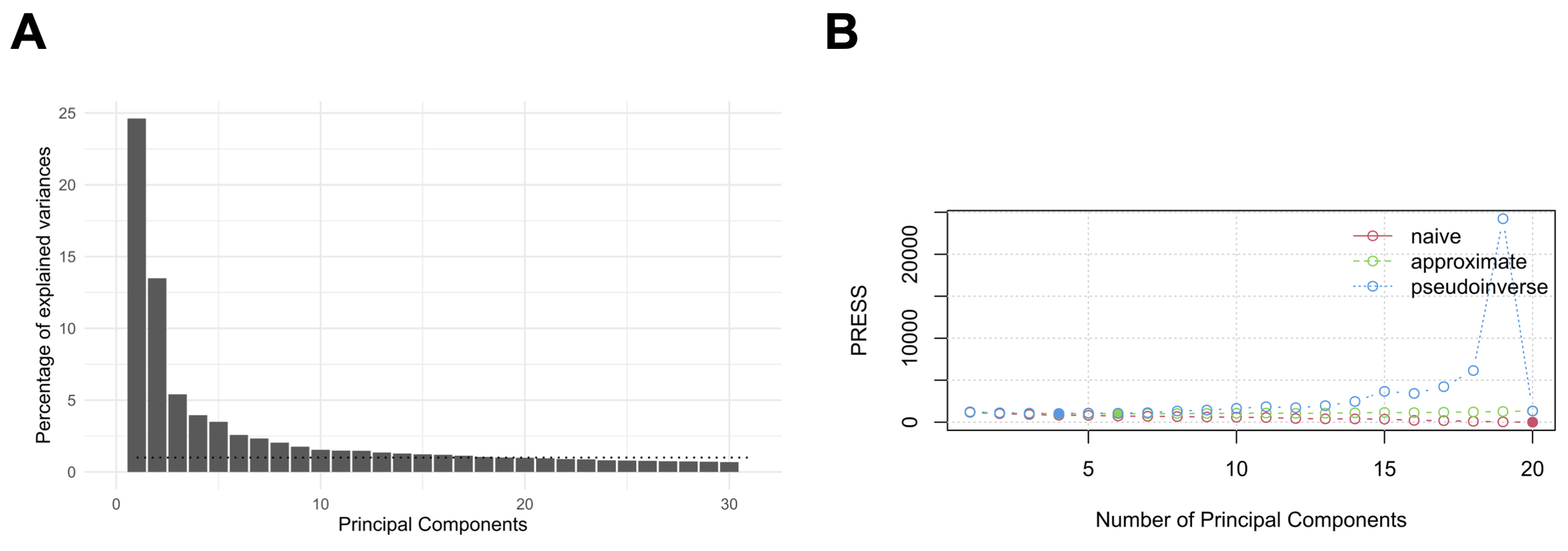

Supplement: S1 Fig — (A) Elbow plot for first 30 Principal Components (PCs). Dotted line represents the cut-off of 1% explained variance, crossing between PC 19 and 20. (B) Predicted sum of squares (PRESS) vs. number of PCs for first 20 PCs. Solid dot represents minimum value of PRESS. (TIF) [file pcbi.1010204.s001.tif]

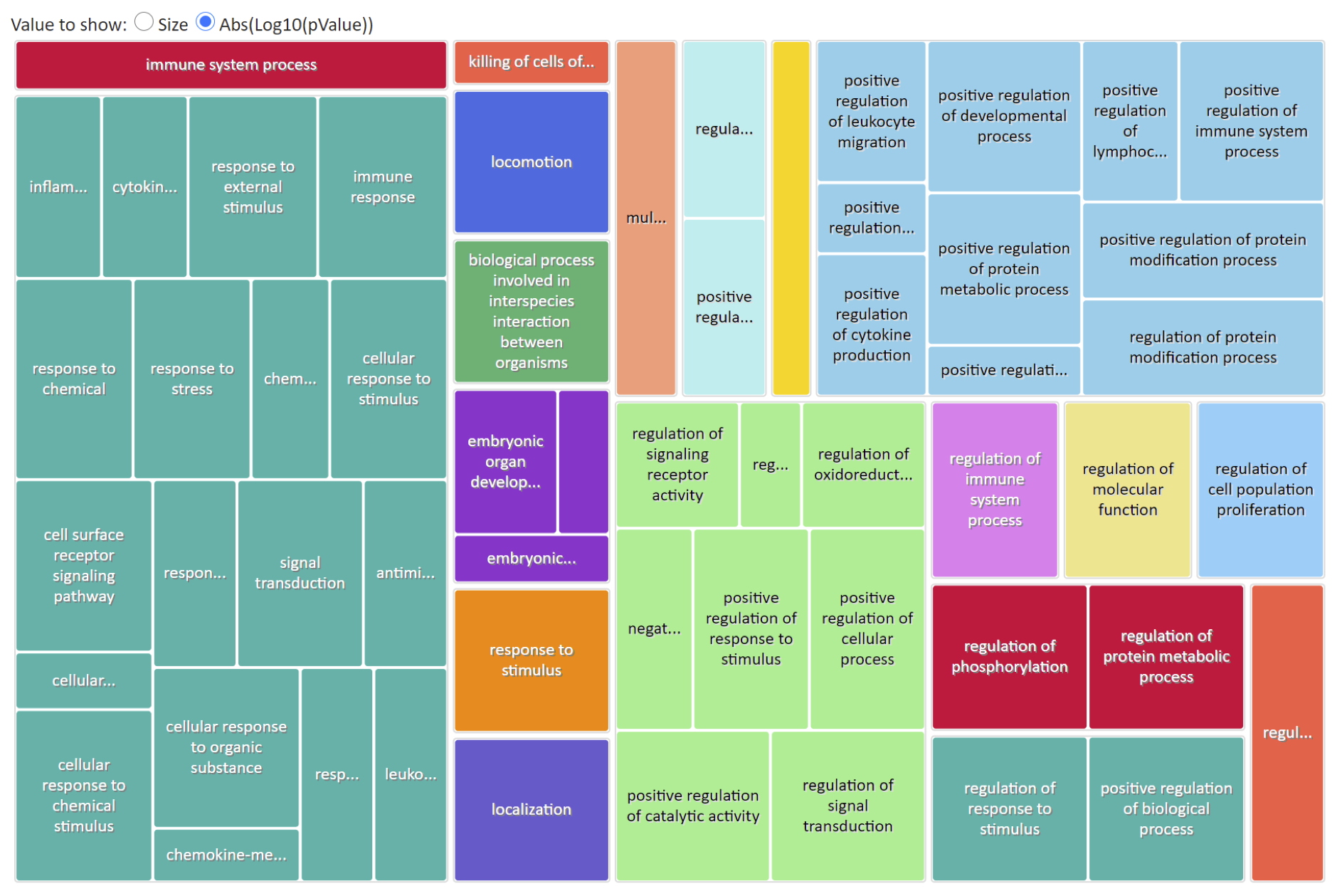

Supplement: S2 Fig — Size of each rectangle represents log10 p-value of the GO terms. (TIF) [file pcbi.1010204.s002.tif]
