# Supplementary material for: ATRPred: A machine learning based tool for clinical decision making of anti-TNF treatment in rheumatoid arthritis patients
Source: PLoS Comput Biol. 2022 Jul 5;18(7):e1010204. doi: 10.1371/journal.pcbi.1010204 (PMC9321399; doi:10.1371/journal.pcbi.1010204)
Supplement: S1 Table — The best model performance with 17 protein features along with baseline DAS and gender information is highlighted in grey. (DOCX) [file pcbi.1010204.s003.docx]

**S1 Table.** The ML classifier performance with 5-fold nested cross validation and the inclusion of protein features one-by-one with decreasing feature importance along with baseline DAS and gender information. The best model performance with 17 protein features along with baseline DAS and gender information is highlighted in grey.

| **No. of proteins in the feature set** | **5-fold Mean AUC** | | **5-fold Mean Accuracy** | | **5-fold Mean Sensitivity** | | **5-fold Mean Specificity** | | **5-fold Mean MCC** | |
| --- | --- | --- | --- | --- | --- | --- | --- | --- | --- | --- |
|  | ***Train*** | ***Test*** | ***Train*** | ***Test*** | ***Train*** | ***Test*** | ***Train*** | ***Test*** | ***Train*** | ***Test*** |
| 0 | 0.59 | 0.57 | 0.61 | 0.5 | 0.51 | 0.36 | 0.65 | 0.59 | 0.17 | -0.05 |
| 1 | 0.68 | 0.58 | 0.67 | 0.54 | 0.66 | 0.48 | 0.68 | 0.58 | 0.32 | 0.06 |
| 2 | 0.73 | 0.73 | 0.71 | 0.66 | 0.68 | 0.67 | 0.73 | 0.71 | 0.4 | 0.32 |
| 3 | 0.83 | 0.77 | 0.79 | 0.68 | 0.81 | 0.69 | 0.78 | 0.69 | 0.56 | 0.35 |
| 4 | 0.85 | 0.77 | 0.81 | 0.66 | 0.78 | 0.7 | 0.82 | 0.64 | 0.6 | 0.32 |
| 5 | 0.87 | 0.77 | 0.82 | 0.7 | 0.81 | 0.67 | 0.82 | 0.72 | 0.62 | 0.4 |
| 6 | 0.88 | 0.79 | 0.84 | 0.68 | 0.79 | 0.65 | 0.86 | 0.72 | 0.64 | 0.39 |
| 7 | 0.89 | 0.86 | 0.88 | 0.75 | 0.77 | 0.59 | 0.94 | 0.85 | 0.73 | 0.51 |
| 8 | 0.91 | 0.78 | 0.84 | 0.71 | 0.92 | 0.69 | 0.81 | 0.7 | 0.69 | 0.38 |
| 9 | 0.91 | 0.8 | 0.86 | 0.71 | 0.84 | 0.63 | 0.87 | 0.76 | 0.7 | 0.39 |
| 10 | 0.84 | 0.74 | 0.79 | 0.69 | 0.79 | 0.61 | 0.8 | 0.71 | 0.57 | 0.33 |
| 11 | 0.93 | 0.79 | 0.88 | 0.73 | 0.86 | 0.62 | 0.89 | 0.8 | 0.75 | 0.43 |
| 12 | 0.94 | 0.79 | 0.87 | 0.72 | 0.9 | 0.64 | 0.86 | 0.77 | 0.74 | 0.4 |
| 13 | 0.93 | 0.73 | 0.88 | 0.7 | 0.89 | 0.57 | 0.87 | 0.76 | 0.74 | 0.32 |
| 14 | 0.95 | 0.72 | 0.9 | 0.77 | 0.93 | 0.69 | 0.88 | 0.81 | 0.79 | 0.47 |
| 15 | 0.95 | 0.75 | 0.89 | 0.67 | 0.93 | 0.66 | 0.86 | 0.68 | 0.77 | 0.31 |
| 16 | 0.96 | 0.73 | 0.9 | 0.65 | 0.9 | 0.51 | 0.9 | 0.72 | 0.79 | 0.22 |
| 17 | 0.99 | 0.86 | 0.95 | 0.81 | 0.98 | 0.75 | 0.93 | 0.86 | 0.89 | 0.6 |
| 18 | 0.99 | 0.81 | 0.97 | 0.75 | 0.98 | 0.66 | 0.96 | 0.8 | 0.93 | 0.44 |
| 19 | 0.98 | 0.79 | 0.95 | 0.73 | 0.97 | 0.72 | 0.94 | 0.74 | 0.89 | 0.43 |
| 20 | 1 | 0.86 | 0.98 | 0.78 | 0.99 | 0.65 | 0.97 | 0.83 | 0.95 | 0.51 |
| 21 | 1 | 0.8 | 0.98 | 0.75 | 0.98 | 0.59 | 0.97 | 0.85 | 0.95 | 0.44 |
| 22 | 0.99 | 0.81 | 0.97 | 0.75 | 0.97 | 0.58 | 0.96 | 0.86 | 0.93 | 0.47 |
| 23 | 0.99 | 0.79 | 0.96 | 0.75 | 0.99 | 0.59 | 0.95 | 0.84 | 0.92 | 0.43 |
| 24 | 1 | 0.83 | 1 | 0.78 | 1 | 0.53 | 1 | 0.84 | 0.99 | 0.45 |
| 25 | 1 | 0.78 | 0.99 | 0.72 | 0.97 | 0.58 | 1 | 0.82 | 0.98 | 0.36 |
| 26 | 0.99 | 0.86 | 0.98 | 0.74 | 0.98 | 0.5 | 0.98 | 0.85 | 0.96 | 0.37 |
| 27 | 1 | 0.79 | 0.99 | 0.71 | 0.99 | 0.52 | 1 | 0.8 | 0.99 | 0.34 |
| 28 | 1 | 0.77 | 1 | 0.73 | 1 | 0.43 | 1 | 0.86 | 1 | 0.34 |
| 29 | 0.99 | 0.73 | 0.98 | 0.67 | 0.97 | 0.47 | 0.99 | 0.77 | 0.96 | 0.23 |
| 30 | 1 | 0.74 | 0.99 | 0.71 | 1 | 0.57 | 0.99 | 0.8 | 0.98 | 0.38 |
