# Supplementary material for: ATRPred: A machine learning based tool for clinical decision making of anti-TNF treatment in rheumatoid arthritis patients
Source: PLoS Comput Biol. 2022 Jul 5;18(7):e1010204. doi: 10.1371/journal.pcbi.1010204 (PMC9321399; doi:10.1371/journal.pcbi.1010204)
Supplement: S2 Table — (DOCX) [file pcbi.1010204.s004.docx]

**S2 Table.** Enrichment analysis of Gene Ontology terms (Biological Process).

| **GO term ID** | **Term description** | **Observed gene count** | **Background gene count** | **Percentage** | **False discovery rate** | **Matching proteins in your network (IDs)** | **Matching proteins in your network (labels)** |
| --- | --- | --- | --- | --- | --- | --- | --- |
| GO:0006954 | inflammatory response | 6 | 482 | 1.24% | 0.0024 | ENSP00000263125,ENSP00000276431,ENSP00000304915,ENSP00000378118,ENSP00000379110,ENSP00000418009 | CCL8,CXCL1,IL13,PRKCQ,RARRES2,TNFRSF10B |
| GO:0002684 | positive regulation of immune system process | 7 | 882 | 0.79% | 0.0027 | ENSP00000263125,ENSP00000304915,ENSP00000351407,ENSP00000365048,ENSP00000378118,ENSP00000379110,ENSP00000418009 | ARNT,CCL8,CXCL1,IL13,PRKCQ,RARRES2,TNFSF13B |
| GO:0002682 | regulation of immune system process | 8 | 1391 | 0.58% | 0.0032 | ENSP00000263125,ENSP00000304915,ENSP00000351407,ENSP00000365048,ENSP00000378118,ENSP00000379110,ENSP00000418009,ENSP00000479089 | ARNT,CCL8,CXCL1,IL13,OSCAR,PRKCQ,RARRES2,TNFSF13B |
| GO:0019221 | cytokine-mediated signaling pathway | 6 | 655 | 0.92% | 0.0034 | ENSP00000276431,ENSP00000304915,ENSP00000322788,ENSP00000365048,ENSP00000378118,ENSP00000379110 | CCL8,CXCL1,IL13,MMP1,TNFRSF10B,TNFSF13B |
| GO:0007166 | cell surface receptor signaling pathway | 9 | 2198 | 0.41% | 0.0037 | ENSP00000263125,ENSP00000276431,ENSP00000304915,ENSP00000322788,ENSP00000355124,ENSP00000365048,ENSP00000378118,ENSP00000379110,ENSP00000479089 | CCL8,CXCL1,IL13,KRT19,MMP1,OSCAR,PRKCQ,TNFRSF10B,TNFSF13B |
| GO:0040011 | locomotion | 7 | 1144 | 0.61% | 0.0037 | ENSP00000263125,ENSP00000276431,ENSP00000322788,ENSP00000378118,ENSP00000379110,ENSP00000409007,ENSP00000418009 | CCL8,CXCL1,GDNF,MMP1,PRKCQ,RARRES2,TNFRSF10B |
| GO:0042127 | regulation of cell population proliferation | 8 | 1594 | 0.50% | 0.0037 | ENSP00000263125,ENSP00000276431,ENSP00000304915,ENSP00000351407,ENSP00000365048,ENSP00000378118,ENSP00000379110,ENSP00000409007 | ARNT,CCL8,CXCL1,GDNF,IL13,PRKCQ,TNFRSF10B,TNFSF13B |
| GO:0048584 | positive regulation of response to stimulus | 9 | 2054 | 0.44% | 0.0037 | ENSP00000263125,ENSP00000276431,ENSP00000304915,ENSP00000351407,ENSP00000365048,ENSP00000378118,ENSP00000379110,ENSP00000409007,ENSP00000418009 | ARNT,CCL8,CXCL1,GDNF,IL13,PRKCQ,RARRES2,TNFRSF10B,TNFSF13B |
| GO:0070663 | regulation of leukocyte proliferation | 4 | 213 | 1.88% | 0.0037 | ENSP00000263125,ENSP00000304915,ENSP00000365048,ENSP00000378118 | CCL8,IL13,PRKCQ,TNFSF13B |
| GO:0070887 | cellular response to chemical stimulus | 10 | 2672 | 0.37% | 0.0037 | ENSP00000263125,ENSP00000272190,ENSP00000276431,ENSP00000304915,ENSP00000322788,ENSP00000351407,ENSP00000365048,ENSP00000378118,ENSP00000379110,ENSP00000409007 | ARNT,CCL8,CXCL1,GDNF,IL13,MMP1,PRKCQ,REN,TNFRSF10B,TNFSF13B |
| GO:0016477 | cell migration | 6 | 812 | 0.74% | 0.0042 | ENSP00000263125,ENSP00000276431,ENSP00000322788,ENSP00000378118,ENSP00000379110,ENSP00000409007 | CCL8,CXCL1,GDNF,MMP1,PRKCQ,TNFRSF10B |
| GO:0002376 | immune system process | 9 | 2370 | 0.38% | 0.0043 | ENSP00000263125,ENSP00000276431,ENSP00000304915,ENSP00000322788,ENSP00000365048,ENSP00000378118,ENSP00000379110,ENSP00000418009,ENSP00000479089 | CCL8,CXCL1,IL13,MMP1,OSCAR,PRKCQ,RARRES2,TNFRSF10B,TNFSF13B |
| GO:0006935 | chemotaxis | 5 | 491 | 1.02% | 0.0043 | ENSP00000263125,ENSP00000378118,ENSP00000379110,ENSP00000409007,ENSP00000418009 | CCL8,CXCL1,GDNF,PRKCQ,RARRES2 |
| GO:0009605 | response to external stimulus | 8 | 1857 | 0.43% | 0.0043 | ENSP00000263125,ENSP00000272190,ENSP00000276431,ENSP00000304915,ENSP00000378118,ENSP00000379110,ENSP00000409007,ENSP00000418009 | CCL8,CXCL1,GDNF,IL13,PRKCQ,RARRES2,REN,TNFRSF10B |
| GO:0048583 | regulation of response to stimulus | 11 | 3882 | 0.28% | 0.0043 | ENSP00000263125,ENSP00000272190,ENSP00000276431,ENSP00000304915,ENSP00000351407,ENSP00000365048,ENSP00000378118,ENSP00000379110,ENSP00000409007,ENSP00000418009,ENSP00000479089 | ARNT,CCL8,CXCL1,GDNF,IL13,OSCAR,PRKCQ,RARRES2,REN,TNFRSF10B,TNFSF13B |
| GO:0051707 | response to other organism | 6 | 835 | 0.72% | 0.0043 | ENSP00000272190,ENSP00000276431,ENSP00000304915,ENSP00000378118,ENSP00000379110,ENSP00000418009 | CCL8,CXCL1,IL13,RARRES2,REN,TNFRSF10B |
| GO:0009617 | response to bacterium | 5 | 555 | 0.90% | 0.0044 | ENSP00000272190,ENSP00000276431,ENSP00000304915,ENSP00000379110,ENSP00000418009 | CXCL1,IL13,RARRES2,REN,TNFRSF10B |
| GO:0010469 | regulation of signaling receptor activity | 5 | 577 | 0.87% | 0.0048 | ENSP00000304915,ENSP00000365048,ENSP00000378118,ENSP00000379110,ENSP00000409007 | CCL8,CXCL1,GDNF,IL13,TNFSF13B |
| GO:0032496 | response to lipopolysaccharide | 4 | 298 | 1.34% | 0.0048 | ENSP00000272190,ENSP00000276431,ENSP00000304915,ENSP00000379110 | CXCL1,IL13,REN,TNFRSF10B |
| GO:0042221 | response to chemical | 11 | 4153 | 0.26% | 0.0055 | ENSP00000263125,ENSP00000272190,ENSP00000276431,ENSP00000304915,ENSP00000322788,ENSP00000351407,ENSP00000365048,ENSP00000378118,ENSP00000379110,ENSP00000409007,ENSP00000418009 | ARNT,CCL8,CXCL1,GDNF,IL13,MMP1,PRKCQ,RARRES2,REN,TNFRSF10B,TNFSF13B |
| GO:0002687 | positive regulation of leukocyte migration | 3 | 127 | 2.36% | 0.0066 | ENSP00000378118,ENSP00000379110,ENSP00000418009 | CCL8,CXCL1,RARRES2 |
| GO:0006955 | immune response | 7 | 1560 | 0.45% | 0.0069 | ENSP00000276431,ENSP00000304915,ENSP00000365048,ENSP00000378118,ENSP00000379110,ENSP00000418009,ENSP00000479089 | CCL8,CXCL1,IL13,OSCAR,RARRES2,TNFRSF10B,TNFSF13B |
| GO:0050671 | positive regulation of lymphocyte proliferation | 3 | 130 | 2.31% | 0.0069 | ENSP00000263125,ENSP00000304915,ENSP00000365048 | IL13,PRKCQ,TNFSF13B |
| GO:0019730 | antimicrobial humoral response | 3 | 143 | 2.10% | 0.008 | ENSP00000378118,ENSP00000379110,ENSP00000418009 | CCL8,CXCL1,RARRES2 |
| GO:0051704 | multi-organism process | 8 | 2222 | 0.36% | 0.008 | ENSP00000272190,ENSP00000276431,ENSP00000304915,ENSP00000322788,ENSP00000355124,ENSP00000378118,ENSP00000379110,ENSP00000418009 | CCL8,CXCL1,IL13,KRT19,MMP1,RARRES2,REN,TNFRSF10B |
| GO:0009966 | regulation of signal transduction | 9 | 3033 | 0.30% | 0.0113 | ENSP00000263125,ENSP00000272190,ENSP00000276431,ENSP00000304915,ENSP00000351407,ENSP00000365048,ENSP00000378118,ENSP00000379110,ENSP00000409007 | ARNT,CCL8,CXCL1,GDNF,IL13,PRKCQ,REN,TNFRSF10B,TNFSF13B |
| GO:0048568 | embryonic organ development | 4 | 417 | 0.96% | 0.0115 | ENSP00000351407,ENSP00000355124,ENSP00000409007,ENSP00000418009 | ARNT,GDNF,KRT19,RARRES2 |
| GO:0007165 | signal transduction | 11 | 4738 | 0.23% | 0.0123 | ENSP00000263125,ENSP00000272190,ENSP00000276431,ENSP00000304915,ENSP00000322788,ENSP00000355124,ENSP00000365048,ENSP00000378118,ENSP00000379110,ENSP00000409007,ENSP00000479089 | CCL8,CXCL1,GDNF,IL13,KRT19,MMP1,OSCAR,PRKCQ,REN,TNFRSF10B,TNFSF13B |
| GO:0050896 | response to stimulus | 14 | 7824 | 0.18% | 0.0123 | ENSP00000263125,ENSP00000272190,ENSP00000276431,ENSP00000304915,ENSP00000322788,ENSP00000351407,ENSP00000355124,ENSP00000365048,ENSP00000368066,ENSP00000378118,ENSP00000379110,ENSP00000409007,ENSP00000418009,ENSP00000479089 | ARNT,CCL8,CXCL1,GDNF,HAO1,IL13,KRT19,MMP1,OSCAR,PRKCQ,RARRES2,REN,TNFRSF10B,TNFSF13B |
| GO:0060326 | cell chemotaxis | 3 | 183 | 1.64% | 0.0131 | ENSP00000263125,ENSP00000378118,ENSP00000379110 | CCL8,CXCL1,PRKCQ |
| GO:0030890 | positive regulation of B cell proliferation | 2 | 42 | 4.76% | 0.0158 | ENSP00000304915,ENSP00000365048 | IL13,TNFSF13B |
| GO:0071356 | cellular response to tumor necrosis factor | 3 | 197 | 1.52% | 0.0158 | ENSP00000276431,ENSP00000365048,ENSP00000378118 | CCL8,TNFRSF10B,TNFSF13B |
| GO:0006950 | response to stress | 9 | 3267 | 0.28% | 0.016 | ENSP00000263125,ENSP00000272190,ENSP00000276431,ENSP00000304915,ENSP00000351407,ENSP00000368066,ENSP00000378118,ENSP00000379110,ENSP00000418009 | ARNT,CCL8,CXCL1,HAO1,IL13,PRKCQ,RARRES2,REN,TNFRSF10B |
| GO:0008284 | positive regulation of cell population proliferation | 5 | 878 | 0.57% | 0.0163 | ENSP00000263125,ENSP00000304915,ENSP00000351407,ENSP00000365048,ENSP00000409007 | ARNT,GDNF,IL13,PRKCQ,TNFSF13B |
| GO:0071622 | regulation of granulocyte chemotaxis | 2 | 47 | 4.26% | 0.0169 | ENSP00000379110,ENSP00000418009 | CXCL1,RARRES2 |
| GO:0032940 | secretion by cell | 5 | 959 | 0.52% | 0.0206 | ENSP00000365048,ENSP00000378118,ENSP00000379110,ENSP00000418009,ENSP00000479089 | CCL8,CXCL1,OSCAR,RARRES2,TNFSF13B |
| GO:0042325 | regulation of phosphorylation | 6 | 1465 | 0.41% | 0.0206 | ENSP00000272190,ENSP00000276431,ENSP00000304915,ENSP00000351407,ENSP00000378118,ENSP00000418009 | ARNT,CCL8,IL13,RARRES2,REN,TNFRSF10B |
| GO:0051179 | localization | 11 | 5233 | 0.21% | 0.0213 | ENSP00000263125,ENSP00000276431,ENSP00000322788,ENSP00000365048,ENSP00000368066,ENSP00000376855,ENSP00000378118,ENSP00000379110,ENSP00000409007,ENSP00000418009,ENSP00000479089 | CCL8,CXCL1,DPP10,GDNF,HAO1,MMP1,OSCAR,PRKCQ,RARRES2,TNFRSF10B,TNFSF13B |
| GO:0051716 | cellular response to stimulus | 12 | 6212 | 0.19% | 0.0213 | ENSP00000263125,ENSP00000272190,ENSP00000276431,ENSP00000304915,ENSP00000322788,ENSP00000351407,ENSP00000355124,ENSP00000365048,ENSP00000378118,ENSP00000379110,ENSP00000409007,ENSP00000479089 | ARNT,CCL8,CXCL1,GDNF,IL13,KRT19,MMP1,OSCAR,PRKCQ,REN,TNFRSF10B,TNFSF13B |
| GO:0042327 | positive regulation of phosphorylation | 5 | 984 | 0.51% | 0.0214 | ENSP00000276431,ENSP00000304915,ENSP00000351407,ENSP00000378118,ENSP00000418009 | ARNT,CCL8,IL13,RARRES2,TNFRSF10B |
| GO:0030858 | positive regulation of epithelial cell differentiation | 2 | 59 | 3.39% | 0.0219 | ENSP00000304915,ENSP00000409007 | GDNF,IL13 |
| GO:0048518 | positive regulation of biological process | 11 | 5459 | 0.20% | 0.0264 | ENSP00000263125,ENSP00000276431,ENSP00000304915,ENSP00000322788,ENSP00000351407,ENSP00000365048,ENSP00000376855,ENSP00000378118,ENSP00000379110,ENSP00000409007,ENSP00000418009 | ARNT,CCL8,CXCL1,DPP10,GDNF,IL13,MMP1,PRKCQ,RARRES2,TNFRSF10B,TNFSF13B |
| GO:0051247 | positive regulation of protein metabolic process | 6 | 1587 | 0.38% | 0.0264 | ENSP00000263125,ENSP00000276431,ENSP00000304915,ENSP00000351407,ENSP00000378118,ENSP00000418009 | ARNT,CCL8,IL13,PRKCQ,RARRES2,TNFRSF10B |
| GO:0071310 | cellular response to organic substance | 7 | 2219 | 0.32% | 0.0264 | ENSP00000272190,ENSP00000276431,ENSP00000304915,ENSP00000322788,ENSP00000365048,ENSP00000378118,ENSP00000379110 | CCL8,CXCL1,IL13,MMP1,REN,TNFRSF10B,TNFSF13B |
| GO:1900076 | regulation of cellular response to insulin stimulus | 2 | 72 | 2.78% | 0.0276 | ENSP00000263125,ENSP00000418009 | PRKCQ,RARRES2 |
| GO:0050900 | leukocyte migration | 3 | 296 | 1.01% | 0.0294 | ENSP00000276431,ENSP00000322788,ENSP00000378118 | CCL8,MMP1,TNFRSF10B |
| GO:0070098 | chemokine-mediated signaling pathway | 2 | 75 | 2.67% | 0.0294 | ENSP00000378118,ENSP00000379110 | CCL8,CXCL1 |
| GO:0071260 | cellular response to mechanical stimulus | 2 | 78 | 2.56% | 0.0297 | ENSP00000276431,ENSP00000304915 | IL13,TNFRSF10B |
| GO:0001701 | in utero embryonic development | 3 | 306 | 0.98% | 0.0305 | ENSP00000351407,ENSP00000355124,ENSP00000418009 | ARNT,KRT19,RARRES2 |
| GO:0031401 | positive regulation of protein modification process | 5 | 1149 | 0.44% | 0.0305 | ENSP00000276431,ENSP00000304915,ENSP00000351407,ENSP00000378118,ENSP00000418009 | ARNT,CCL8,IL13,RARRES2,TNFRSF10B |
| GO:0033209 | tumor necrosis factor-mediated signaling pathway | 2 | 81 | 2.47% | 0.0305 | ENSP00000276431,ENSP00000365048 | TNFRSF10B,TNFSF13B |
| GO:0001892 | embryonic placenta development | 2 | 86 | 2.33% | 0.0324 | ENSP00000351407,ENSP00000355124 | ARNT,KRT19 |
| GO:0031399 | regulation of protein modification process | 6 | 1747 | 0.34% | 0.0327 | ENSP00000272190,ENSP00000276431,ENSP00000304915,ENSP00000351407,ENSP00000378118,ENSP00000418009 | ARNT,CCL8,IL13,RARRES2,REN,TNFRSF10B |
| GO:0002824 | positive regulation of adaptive immune response based on somatic recombination of immune receptors built from immunoglobulin superfamily domains | 2 | 89 | 2.25% | 0.0338 | ENSP00000263125,ENSP00000365048 | PRKCQ,TNFSF13B |
| GO:0031640 | killing of cells of other organism | 2 | 89 | 2.25% | 0.0338 | ENSP00000378118,ENSP00000379110 | CCL8,CXCL1 |
| GO:0001823 | mesonephros development | 2 | 91 | 2.20% | 0.0341 | ENSP00000272190,ENSP00000409007 | GDNF,REN |
| GO:0002690 | positive regulation of leukocyte chemotaxis | 2 | 91 | 2.20% | 0.0341 | ENSP00000379110,ENSP00000418009 | CXCL1,RARRES2 |
| GO:0042102 | positive regulation of T cell proliferation | 2 | 92 | 2.17% | 0.0341 | ENSP00000263125,ENSP00000365048 | PRKCQ,TNFSF13B |
| GO:0044419 | interspecies interaction between organisms | 4 | 724 | 0.55% | 0.0344 | ENSP00000322788,ENSP00000355124,ENSP00000378118,ENSP00000379110 | CCL8,CXCL1,KRT19,MMP1 |
| GO:0051341 | regulation of oxidoreductase activity | 2 | 93 | 2.15% | 0.0344 | ENSP00000304915,ENSP00000409007 | GDNF,IL13 |
| GO:0048522 | positive regulation of cellular process | 10 | 4898 | 0.20% | 0.0348 | ENSP00000263125,ENSP00000276431,ENSP00000304915,ENSP00000322788,ENSP00000351407,ENSP00000365048,ENSP00000378118,ENSP00000379110,ENSP00000409007,ENSP00000418009 | ARNT,CCL8,CXCL1,GDNF,IL13,MMP1,PRKCQ,RARRES2,TNFRSF10B,TNFSF13B |
| GO:0032879 | regulation of localization | 7 | 2524 | 0.28% | 0.0389 | ENSP00000272190,ENSP00000304915,ENSP00000376855,ENSP00000378118,ENSP00000379110,ENSP00000409007,ENSP00000418009 | CCL8,CXCL1,DPP10,GDNF,IL13,RARRES2,REN |
| GO:2001237 | negative regulation of extrinsic apoptotic signaling pathway | 2 | 104 | 1.92% | 0.0405 | ENSP00000276431,ENSP00000409007 | GDNF,TNFRSF10B |
| GO:0006887 | exocytosis | 4 | 774 | 0.52% | 0.0416 | ENSP00000378118,ENSP00000379110,ENSP00000418009,ENSP00000479089 | CCL8,CXCL1,OSCAR,RARRES2 |
| GO:0051094 | positive regulation of developmental process | 5 | 1286 | 0.39% | 0.0416 | ENSP00000304915,ENSP00000351407,ENSP00000365048,ENSP00000409007,ENSP00000418009 | ARNT,GDNF,IL13,RARRES2,TNFSF13B |
| GO:0061844 | antimicrobial humoral immune response mediated by antimicrobial peptide | 2 | 107 | 1.87% | 0.0416 | ENSP00000378118,ENSP00000379110 | CCL8,CXCL1 |
| GO:0065009 | regulation of molecular function | 8 | 3322 | 0.24% | 0.0416 | ENSP00000263125,ENSP00000276431,ENSP00000304915,ENSP00000365048,ENSP00000376855,ENSP00000378118,ENSP00000379110,ENSP00000409007 | CCL8,CXCL1,DPP10,GDNF,IL13,PRKCQ,TNFRSF10B,TNFSF13B |
| GO:0001819 | positive regulation of cytokine production | 3 | 390 | 0.77% | 0.0451 | ENSP00000263125,ENSP00000304915,ENSP00000351407 | ARNT,IL13,PRKCQ |
| GO:0048608 | reproductive structure development | 3 | 405 | 0.74% | 0.0483 | ENSP00000272190,ENSP00000351407,ENSP00000355124 | ARNT,KRT19,REN |
| GO:0051246 | regulation of protein metabolic process | 7 | 2668 | 0.26% | 0.0483 | ENSP00000263125,ENSP00000272190,ENSP00000276431,ENSP00000304915,ENSP00000351407,ENSP00000378118,ENSP00000418009 | ARNT,CCL8,IL13,PRKCQ,RARRES2,REN,TNFRSF10B |
| GO:0001932 | regulation of protein phosphorylation | 5 | 1370 | 0.36% | 0.0486 | ENSP00000272190,ENSP00000276431,ENSP00000304915,ENSP00000378118,ENSP00000418009 | CCL8,IL13,RARRES2,REN,TNFRSF10B |
| GO:0043085 | positive regulation of catalytic activity | 5 | 1381 | 0.36% | 0.0499 | ENSP00000263125,ENSP00000276431,ENSP00000378118,ENSP00000379110,ENSP00000409007 | CCL8,CXCL1,GDNF,PRKCQ,TNFRSF10B |
