# Supplementary material for: ATRPred: A machine learning based tool for clinical decision making of anti-TNF treatment in rheumatoid arthritis patients
Source: PLoS Comput Biol. 2022 Jul 5;18(7):e1010204. doi: 10.1371/journal.pcbi.1010204 (PMC9321399; doi:10.1371/journal.pcbi.1010204)
Supplement: S3 Table — (DOCX) [file pcbi.1010204.s005.docx]

**S3 Table.** REVIGO summary analysis of Gene Ontology terms (Biological Process).

| **GO term ID** | **Description** | **Frequency in database** | **log_10_pvalue** | **Uniqueness** | **Dispensability** | **Representative** |
| --- | --- | --- | --- | --- | --- | --- |
| GO:0002376 | immune system process | 0.60% | -2.3665 | 0.972 | 0 | immune system process |
| GO:0002682 | regulation of immune system process | 0.25% | -2.4949 | 0.6 | 0 | regulation of immune system process |
| GO:0042127 | regulation of cell proliferation | 0.31% | -2.4318 | 0.654 | 0.225 | regulation of immune system process |
| GO:0048518 | positive regulation of biological process | 1.74% | -1.5784 | 0.714 | 0.281 | regulation of immune system process |
| GO:0043085 | positive regulation of catalytic activity | 0.82% | -1.3019 | 0.719 | 0.611 | regulation of immune system process |
| GO:0031640 | killing of cells of other organism | 0.02% | -1.4711 | 0.778 | 0.173 | regulation of immune system process |
| GO:0051341 | regulation of oxidoreductase activity | 0.02% | -1.4634 | 0.755 | 0.269 | regulation of immune system process |
| GO:0044419 | interspecies interaction between organisms | 0.26% | -1.4634 | 0.953 | 0.669 | regulation of immune system process |
| GO:0051246 | regulation of protein metabolic process | 1.55% | -1.3161 | 0.698 | 0.393 | regulation of immune system process |
| GO:0042325 | regulation of phosphorylation | 0.47% | -1.6861 | 0.686 | 0.243 | regulation of immune system process |
| GO:0065009 | regulation of molecular function | 1.73% | -1.3809 | 0.725 | 0.311 | regulation of immune system process |
| GO:0032879 | regulation of localization | 0.73% | -1.4101 | 0.702 | 0.295 | regulation of immune system process |
| GO:0006954 | inflammatory response | 0.11% | -2.6198 | 0.752 | 0 | inflammatory response |
| GO:0070887 | cellular response to chemical stimulus | 1.01% | -2.4318 | 0.625 | 0.457 | inflammatory response |
| GO:2001237 | negative regulation of extrinsic apoptotic signaling pathway | 0.02% | -1.3925 | 0.562 | 0.638 | inflammatory response |
| GO:0051707 | response to other organism | 0.30% | -2.3665 | 0.651 | 0.352 | inflammatory response |
| GO:0071260 | cellular response to mechanical stimulus | 0.01% | -1.5272 | 0.707 | 0.566 | inflammatory response |
| GO:0001819 | positive regulation of cytokine production | 0.07% | -1.3458 | 0.568 | 0.629 | inflammatory response |
| GO:0009605 | response to external stimulus | 1.37% | -2.3665 | 0.702 | 0.42 | inflammatory response |
| GO:0010469 | regulation of receptor activity | 0.03% | -2.3188 | 0.563 | 0.649 | inflammatory response |
| GO:0048584 | positive regulation of response to stimulus | 0.46% | -2.4318 | 0.42 | 0.367 | inflammatory response |
| GO:0048583 | regulation of response to stimulus | 1.12% | -2.3665 | 0.561 | 0.402 | inflammatory response |
| GO:0006950 | response to stress | 4.58% | -1.7959 | 0.671 | 0.562 | inflammatory response |
| GO:0007166 | cell surface receptor signaling pathway | 0.92% | -2.4318 | 0.526 | 0.321 | inflammatory response |
| GO:0007165 | signal transduction | 6.62% | -1.9101 | 0.448 | 0.679 | inflammatory response |
| GO:0042221 | response to chemical | 3.07% | -2.2596 | 0.682 | 0.475 | inflammatory response |
| GO:1900076 | regulation of cellular response to insulin stimulus | 0.01% | -1.5591 | 0.546 | 0.631 | inflammatory response |
| GO:0030858 | positive regulation of epithelial cell differentiation | 0.01% | -1.6596 | 0.592 | 0.549 | inflammatory response |
| GO:0019221 | cytokine-mediated signaling pathway | 0.09% | -2.4685 | 0.499 | 0.583 | inflammatory response |
| GO:0016477 | cell migration | 0.29% | -2.3768 | 0.781 | 0 | cell migration |
| GO:0032940 | secretion by cell | 0.76% | -1.6861 | 0.845 | 0.257 | cell migration |
| GO:0040011 | locomotion | 1.00% | -2.4318 | 0.972 | 0 | locomotion |
| GO:0050896 | response to stimulus | 12.21% | -1.9101 | 0.975 | 0 | response to stimulus |
| GO:0051179 | localization | 18.50% | -1.6716 | 0.977 | 0 | localization |
| GO:0051704 | multi-organism process | 0.75% | -2.0969 | 0.972 | 0 | multi-organism process |
| GO:0048568 | embryonic organ development | 0.11% | -1.9393 | 0.816 | 0.065 | embryonic organ development |
| GO:0001823 | mesonephros development | 0.02% | -1.4672 | 0.839 | 0.631 | embryonic organ development |
