# Supplementary material for: ATRPred: A machine learning based tool for clinical decision making of anti-TNF treatment in rheumatoid arthritis patients
Source: PLoS Comput Biol. 2022 Jul 5;18(7):e1010204. doi: 10.1371/journal.pcbi.1010204 (PMC9321399; doi:10.1371/journal.pcbi.1010204)
Supplement: S4 Table — (DOCX) [file pcbi.1010204.s006.docx]

**S4 Table.** Enrichment analysis of Gene Ontology terms (Molecular Function).

| **GO term ID** | **Term description** | **Observed gene count** | **Background gene count** | **Percentage** | **False discovery rate** | **Matching proteins in your network (IDs)** | **Matching proteins in your network (labels)** |
| --- | --- | --- | --- | --- | --- | --- | --- |
| GO:0005102 | signaling receptor binding | 10 | 1513 | 0.66% | 1.14E-05 | ENSP00000272190,ENSP00000304915,ENSP00000351407,ENSP00000357086,ENSP00000365048,ENSP00000368066,ENSP00000378118,ENSP00000379110,ENSP00000409007,ENSP00000418009 | ARNT,CCL8,CXCL1,FCRL6,GDNF,HAO1,IL13,RARRES2,REN,TNFSF13B |
| GO:0005125 | cytokine activity | 4 | 216 | 1.85% | 0.002 | ENSP00000304915,ENSP00000365048,ENSP00000378118,ENSP00000379110 | CCL8,CXCL1,IL13,TNFSF13B |
| GO:0005126 | cytokine receptor binding | 4 | 272 | 1.47% | 0.002 | ENSP00000304915,ENSP00000365048,ENSP00000378118,ENSP00000379110 | CCL8,CXCL1,IL13,TNFSF13B |
| GO:0048018 | receptor ligand activity | 5 | 458 | 1.09% | 0.002 | ENSP00000304915,ENSP00000365048,ENSP00000378118,ENSP00000379110,ENSP00000409007 | CCL8,CXCL1,GDNF,IL13,TNFSF13B |
| GO:0008009 | chemokine activity | 2 | 48 | 4.17% | 0.0175 | ENSP00000378118,ENSP00000379110 | CCL8,CXCL1 |
| GO:0005515 | protein binding | 12 | 6605 | 0.18% | 0.0331 | ENSP00000272190,ENSP00000276431,ENSP00000304915,ENSP00000351407,ENSP00000357086,ENSP00000365048,ENSP00000368066,ENSP00000376855,ENSP00000378118,ENSP00000379110,ENSP00000409007,ENSP00000418009 | ARNT,CCL8,CXCL1,DPP10,FCRL6,GDNF,HAO1,IL13,RARRES2,REN,TNFRSF10B,TNFSF13B |
| GO:0005488 | binding | 16 | 11878 | 0.13% | 0.0343 | ENSP00000263125,ENSP00000272190,ENSP00000276431,ENSP00000304915,ENSP00000322788,ENSP00000351407,ENSP00000355124,ENSP00000357086,ENSP00000365048,ENSP00000368066,ENSP00000376855,ENSP00000378118,ENSP00000379110,ENSP00000409007,ENSP00000418009,ENSP00000460236 | ARNT,CCL8,CXCL1,DPP10,FCRL6,GDNF,HAO1,IL13,KRT19,MMP1,PRKCQ,RARRES2,REN,SPON1,TNFRSF10B,TNFSF13B |
| GO:0098772 | molecular function regulator | 6 | 1793 | 0.33% | 0.0376 | ENSP00000304915,ENSP00000365048,ENSP00000376855,ENSP00000378118,ENSP00000379110,ENSP00000409007 | CCL8,CXCL1,DPP10,GDNF,IL13,TNFSF13B |
