# Supplementary material for: ATRPred: A machine learning based tool for clinical decision making of anti-TNF treatment in rheumatoid arthritis patients
Source: PLoS Comput Biol. 2022 Jul 5;18(7):e1010204. doi: 10.1371/journal.pcbi.1010204 (PMC9321399; doi:10.1371/journal.pcbi.1010204)
Supplement: S5 Table — (DOCX) [file pcbi.1010204.s007.docx]

**S5 Table.** Enrichment analysis of Gene Ontology terms (Cellular Component).

| **GO term ID** | **Term description** | **Observed gene count** | **Background gene count** | **Percentage** | **False discovery rate** | **Matching proteins in your network (IDs)** | **Matching proteins in your network (labels)** |
| --- | --- | --- | --- | --- | --- | --- | --- |
| GO:0005576 | extracellular region | 9 | 2505 | 0.36% | 0.0095 | ENSP00000272190,ENSP00000304915,ENSP00000322788,ENSP00000365048,ENSP00000378118,ENSP00000379110,ENSP00000409007,ENSP00000418009,ENSP00000479089 | CCL8,CXCL1,GDNF,IL13,MMP1,OSCAR,RARRES2,REN,TNFSF13B |
| GO:0044421 | extracellular region part | 7 | 1375 | 0.51% | 0.0095 | ENSP00000272190,ENSP00000304915,ENSP00000322788,ENSP00000365048,ENSP00000378118,ENSP00000379110,ENSP00000418009 | CCL8,CXCL1,IL13,MMP1,RARRES2,REN,TNFSF13B |
| GO:0005615 | extracellular space | 6 | 1134 | 0.53% | 0.0101 | ENSP00000272190,ENSP00000304915,ENSP00000365048,ENSP00000378118,ENSP00000379110,ENSP00000418009 | CCL8,CXCL1,IL13,RARRES2,REN,TNFSF13B |
| GO:1904724 | tertiary granule lumen | 2 | 55 | 3.64% | 0.0305 | ENSP00000379110,ENSP00000479089 | CXCL1,OSCAR |
| GO:0035580 | specific granule lumen | 2 | 62 | 3.23% | 0.0308 | ENSP00000379110,ENSP00000479089 | CXCL1,OSCAR |
| GO:0034774 | secretory granule lumen | 3 | 323 | 0.93% | 0.0484 | ENSP00000379110,ENSP00000418009,ENSP00000479089 | CXCL1,OSCAR,RARRES2 |
