# Supplementary material for: ATRPred: A machine learning based tool for clinical decision making of anti-TNF treatment in rheumatoid arthritis patients
Source: PLoS Comput Biol. 2022 Jul 5;18(7):e1010204. doi: 10.1371/journal.pcbi.1010204 (PMC9321399; doi:10.1371/journal.pcbi.1010204)
Supplement: S6 Table — (DOCX) [file pcbi.1010204.s008.docx]

**S6 Table.** Enrichment analysis of KEGG Pathways.

| **GO term ID** | **Term description** | **Observed gene count** | **Background gene count** | **Percentage** | **False discovery rate** | **Matching proteins in your network (IDs)** | **Matching proteins in your network (labels)** |
| --- | --- | --- | --- | --- | --- | --- | --- |
| hsa04060 | Cytokine-cytokine receptor interaction | 5 | 263 | 1.90% | 0.00012 | ENSP00000276431,ENSP00000304915,ENSP00000365048,ENSP00000378118,ENSP00000379110 | CCL8,CXCL1,IL13,TNFRSF10B,TNFSF13B |
| hsa04657 | IL-17 signaling pathway | 3 | 92 | 3.26% | 0.0013 | ENSP00000304915,ENSP00000322788,ENSP00000379110 | CXCL1,IL13,MMP1 |
| hsa05323 | Rheumatoid arthritis | 3 | 84 | 3.57% | 0.0013 | ENSP00000322788,ENSP00000365048,ENSP00000379110 | CXCL1,MMP1,TNFSF13B |
| hsa05162 | Measles | 3 | 133 | 2.26% | 0.0024 | ENSP00000263125,ENSP00000276431,ENSP00000304915 | IL13,PRKCQ,TNFRSF10B |
| hsa04064 | NF-kappa B signaling pathway | 2 | 93 | 2.15% | 0.0255 | ENSP00000263125,ENSP00000365048 | PRKCQ,TNFSF13B |
| hsa04658 | Th1 and Th2 cell differentiation | 2 | 88 | 2.27% | 0.0255 | ENSP00000263125,ENSP00000304915 | IL13,PRKCQ |
