# Supplementary material for: ATRPred: A machine learning based tool for clinical decision making of anti-TNF treatment in rheumatoid arthritis patients
Source: PLoS Comput Biol. 2022 Jul 5;18(7):e1010204. doi: 10.1371/journal.pcbi.1010204 (PMC9321399; doi:10.1371/journal.pcbi.1010204)
Supplement: Attachment — Submitted filename: Reponse_to_reviewer_v11.pdf [file pcbi.1010204.s009.pdf]

Londonderry, 10th April 2022

Dear Dr Manja Marz

We wish to express sincere thanks to the reviewers for their comprehensive, detailed, and insightful comments. We have taken their all observations on board, and we truly believe that their input has greatly added to the quality of this manuscript. Please see below our point-by-point response to reviewers' comments. While addressing their comments, we have also done some additional analysis. Please see new supplementary figure 1 and updated table 1. We have now included all the R scripts and raw data used in the analysis and development of ATRPred in our Github repository. Following the journal guidelines, the changes made in the manuscript to address reviewers' comments have been highlighted in yellow.

We hope you and all the reviewers are satisfied with the revised version of this manuscript.

Thank you very much for your consideration.

Sincerely
Priyank Shukla, PhD

## Response to reviewers' comments

## Reviewer 1:

**Comment 1:** This is a nicely written prediction study, where a machine learning model used clinical and proteomics data to predict response to TNF blockers after 6 months in patients with rheumatoid arthritis. 89 patients were included. Features included demographic and clinical data and over 300 proteins that have been obtained from 60 responders and 29 non-responders. The form of the manuscript including the figures is clear.

**Response 1:** Authors would like to thank Reviewer 1 for the positive comments.

**Comment 2:** There are some minor limitations that need to be discussed. Whilst the proteomics part is described in detail, the manuscript only provides minimal clinical information. BLDAS and gender are the most important features in the machine learning model. Why is gender so important? How about other features such as rheumatoid factor and or anti-CCP?

**Response 2:** Authors would like to thank the reviewer for vouching for our detailed proteomics. As far as clinical data is concerned, we did have information about RF, anti-CCP/ACPA. But upon analysis none of them turned out to be important or significant feature. We decided to exclude these, as the focus of our paper is on building a machine learning based anti-TNF response

predictor. But following the reviewer's suggestion we have now included these results in updated Table 1. Please see highlighted rows of updated Table 1 in the "changes highlighted" version of the manuscript.

As per updated Table 1, gender and baseline DAS are statistically significant (p-value = 0.006) clinical features, but not the most important compared to Olink proteins signature we identified. This is further confirmed in Supplementary Table 1 as using just baseline DAS and gender we get a very low anti-TNF response predicting power; almost close to random. Furthermore, from Figure 4A, one can see that the contribution (effect size or beta coefficient) of gender is close to 0 (negligible). So, gender was not an important feature independently.

**Comment 3:** Methotrexate is not mentioned once in the text, despite this clearly improves the response rate of TNF blockers. Was this the same in responders and non-responders? Methotrexate might also influence the protein composition in the serum. A section on limitations in the discussion (relatively low patient number, moderate accuracy etc.) would be appropriate.

**Response 3:** We chose to mention conventional DMARD (cDMARD), rather than Methotrexate (MTX), as all 89 patients were previously (before baseline, i.e., before the start of the anti-TNF treatment) on different cDMARDs. Yes, we acknowledge that it is known that MTX or other cDMARDs improve the response rate of anti-TNF. But some of the patients didn't carry onto cDMARD (including MTX) during anti-TNF treatment as they were poorly tolerated or produced adverse reactions prior to anti-TNF. We have now included data of concurrent cDMARD (which includes MTX) in updated Table 1 for both baseline and 6 months. Please see highlighted rows of updated Table 1 in the "changes highlighted" version of the manuscript. Please note that some of them further stopped taking cDMARD at 6 months.

As suggested by the reviewer, we have now mentioned the limitations of the study (relatively low patient number and moderate accuracy) in the discussion section. Please see the highlighted lines 513, 514 and 530 in the discussion section of the "changes highlighted" version of the manuscript.

## <u>Reviewer 2</u>:

**Comment 1:** In the present manuscript, Prasad B et al, use plasma proteomics data from a cohort of rheumatoid arthritis (RA) patients starting anti-TNF therapy. The objective pursued here is of high translational value, since there is yet no tool with which to discriminate responders from non-responder patients to this therapy. However there are several concerning aspects from the actual depth and reproducibility of the study.

**Response 1:** Authors would like to thank the reviewer for commenting on the need for such a tool for clinical usage.

**Comment 2:** Proteomic data: One key concerning aspect is the low number of plasma samples with good proteomic data (89 from 144, 64%). The used platform, OLINK, is a robust proteomic technology and normally yields good protein quantifications. Unless the samples were extremely degraded, usable proteomic data should be obtained from a majority of the patients. Without any convincing explanation on the reason for this issue, it really questions the validity of the remaining proteomic sample data.

**Response 2:** A large number of patients (144 – 89 = 55) were lost to follow-up; either were not able to make 6 months follow-up appointment or couldn't recover, and every composite require to calculate DAS score was not available. Follow-up DAS score was required for knowing a patient's response to anti-TNF and for modelling the ML predictor. Also, not all 144 patients were on ant-TNF; some of them were on other biologic DMARDs (such as Tociluzimab, Ritiuximab, etc). That is why the sample size got reduced for Olink proteomics data analysis. For all the 89 patients' samples for which we had follow-up DAS score, we can confirm that there was not any quality degradation in the protein quantification. So, there was no QC failure for any samples. We have now edited the manuscript to reflect this information. Please see highlighted lines 170-173 in the "changes highlighted" version of the manuscript.

**Comment 3:** Response criteria: two criteria (BSR and NICE) were used to define response. Why two methods? EULAR or ACR response criteria is a more common methods for disease response classification; what was the reason not to use these.

**Response 3:** BSR and NICE criteria are one and the same. We have now removed the mention of NICE from the paper to avoid any confusion. Please see highlighted lines 167-168 in the "changes highlighted" version of the manuscript. Since the patients were recruited in the UK, this was the response criteria used at the time of recruitment.

**Comment 4:** Methodology: The authors describe in detail the bioinformatic packages used and have developed an accompanying R package to apply the methodology, which is useful. However, using the mentioned Github repository, there is substantial missing information describing how to apply the R package. Also, why didn't the authors include the proteomic (and phenotype) data from the study to evaluate the method's performance?

**Response 4:**

How to install and run the ATRPred R package is present in the README file provided at this link of our Github repository: https://github.com/ShuklaLab/ATRPred/blob/main/README.md

We have now included all the R scripts used in the analysis and development of ATRPred in the following folder of our Github repository:
https://github.com/ShuklaLab/ATRPred/tree/main/scripts

We have now included the raw data in the following folder of our Github repository:
https://github.com/ShuklaLab/ATRPred/tree/main/raw_data

Please also see lines 595-597 in the "changes highlighted" version of the manuscript, which reflect the above mentioned updates.

**Comment 5:** Predictor: Internal cross-validation (CV) is used to develop the predictor model. This is a useful approach to estimate the generalizability of the predictor. However, the way that this has been used is highly questionable. Feature selection is done with the entire cohort of patients before CV. This includes a major bias in the procedure since it will increase the overfitting to the current dataset, despite using a cross-validation schema afterwards. Also, it is not clear how the authors balanced the unequal representation of non-responders, which is another potential bias in predictor building.

**Response 5:** Authors thank for pointing out the usefulness of internal CV for generalizability of our ML predictor.

Authors agree that using entire cohort for feature selection can cause bias and therefore overfitting. Since our dataset was small, in the feature selection step (figure 2A middle panel), we went about carrying 500 random simulations (Monte-Carlo) (figure 3A). In each simulation, we randomly selected 80% of our samples for training data and kept aside 20% samples as test data. To make sure that our model does not overfit, in the 5-fold nested cross-validation (figure 2B), we plotted AUC curve for both training set and test set (figure 3B). As a model overfits, performance on training set goes up but the same goes down on the test set. By selecting only 17 protein features, we achieved an optimum model performance (figure 3B). Including more proteins (from the list of important features), would cause model overfitting. Enroth et al. (2019) has applied a similar strategy to identify core proteins for signature.

Authors agree the data imbalance can cause a potential bias for the predictor towards the majority class. If we go about balancing, either we will end up imputing data (usually made our co-author clinicians skeptic) or reducing size (can't do this as our sample size was already small). To address this potential bias, we included a performance metric Matthew's Correlation Coefficient (MCC), which is a good metric in capturing performance even in imbalance sets (Chicco and Jurman, 2020). The value of MCC varies from -1 to 1 and we were able to achieve a value of 0.6 as a performance for our final ML predictor.

References:

1. Enroth S, Berggrund M, Lycke M, Broberg J, Lundberg M, Assarsson E, Olovsson M, Stålberg K, Sundfeldt K, Gyllensten U. (2019). High throughput proteomics identifies a

high-accuracy 11 plasma protein biomarker signature for ovarian cancer. Commun Biol. 2:221. doi: 10.1038/s42003-019-0464-9.
2. Chicco, D., Jurman, G. (2020). The advantages of the Matthews correlation coefficient (MCC) over F1 score and accuracy in binary classification evaluation. BMC Genomics 21, 6. doi: 10.1186/s12864-019-6413-7.

**Comment 6:** The identification of a putative 2 class division of patients is rather arbitrary. First, the separation is done visually, with no assessment of the randomness of this separation. Second, the separating variable, PC3 is not described: how much variance is captured. Third, the fact that is associated to confounders, age and baseline DAS28, clearly does not provide much interest to the finding with regards to the objective at hand.

**Response 6:** We would like to confirm that the two-class division of patients presented in Figure 1 is not arbitrary. Following reviewer's concern, we have now validated our PCA results using leave-one-out cross-validation (LOOCV) method and found the first 4 PCs to be important (new Supplementary Figure 1B). Please also see highlighted line 204-205 and 290-295 in the "changes highlighted" version of the manuscript. The separation seen in PC3 explains only 5.4% variance of the whole Olink proteomics dataset (new Supplementary Figure 1A). We have now also included the variance captured in the Figure 1. Since this separation by PC3 was based on all 352 proteins which were able to explain only 5.4% of variance, we didn't pursue the proteins accounting for this separation. However, the separation made by PC3 was striking, and therefore we further pursued the statistical analysis of clinical features driving those two clusters and found that baseline DAS and gender were in fact significant (Table 2). Literature review indeed suggests these two features are confounders of protein expression; thus, reinforcing our biological understanding. We agree with the reviewer that the 2 classes (endotypes) seen do not provide much interest as they are mainly based on two confounders (gender and BLDAS), as also already discussed in our manuscript. However, we decided to keep this figure 1 in the manuscript, as it reinforces our biological understanding of the data.

In the discussion section of the "changes highlighted" version of our manuscript, we have earlier given following explanation for the relevance of this part of the study:

Line numbers 460-472:
*"The current study uncovered two distinct endotypes based on the expression profile of all 352 plasma proteins, which had significantly different gender proportions and baseline DAS (Figure 1 and Table 2). Since, these endotypes were not significantly different in terms of their anti-TNF treatment response (Table 2), there is a possibility of the existence of two distinct RA disease endotypes, which may be important in other aspects of the disease management or other drug response. Gender is known to be significantly associated with plasma protein profile (Silliman et al., 2012). Further, DAS28 is also known to be correlated with the plasma proteins such as IL37 (Xia et al., 2015) and CXCL10 (Pandya et al., 2017). A significantly higher average ESR has been observed in females of age up to 75 years (Wetteland et al., 1996). Considering the above literature, there is another possibility that the two endotypes uncovered in this study may be*

*totally unrelated to RA. Hence, the clinicians may consider keeping a strict vigil on these endotypes, which may be helpful in better informed decision making."*

Line numbers 575-580:
*"We have identified two clusters (Figure 1 and Table 2) driven by plasma protein profile as a plausible endotypes. Unfortunately, they do not correspond to anti-TNF therapy responsiveness, but they are still significantly different in terms of disease activity and gender, and thus possibly play an important role in patient management. For example, since these endotypes are independent of future treatment response, they may indicate pre-biologic treatment pathology sub-groups, which can be investigated in future studies."*

**Comment 7:** No real test set is used to demonstrate the performance of the method. An application to an independent cohort of patients would really help on the credibility of the proposed patient stratification tool.

**Response 7:** We have used a nested cross-validation scheme, so that the outer cross-validation provides an estimate of the performance, which is not biased by selection of test data. Authors agree with the reviewer for the need of a validation study on a completely independent cohort. Patient recruitment is an expensive and tedious process. However, going forward, we will do a validation study to re-confirm the performance of ATRPred on a completely independent cohort. We plan to apply for research grants based on this current paper to progress our research further. Many papers have published an even smaller sample size for the discovery cohort as compared to ours (N=89). For example, Westwood et al. (2016) reported plasma biomarkers for their discovery cohort (N=79) and later they validated their results in a follow-up study (Westwood et al., 2020).

References:

1. Westwood S, Leoni E, Hye A, Lynham S, Khondoker MR, Ashton NJ, Kiddle SJ, Baird AL, Sainz-Fuertes R, Leung R, Graf J, Hehir CT, Baker D, Cereda C, Bazenet C, Ward M, Thambisetty M, Lovestone S. (2016). Blood-Based Biomarker Candidates of Cerebral Amyloid Using PiB PET in Non-Demented Elderly. J Alzheimers Dis. 52(2):561-72. doi: 10.3233/JAD-151155. PMID: 27031486.
2. Westwood S, Baird AL, Anand SN, Nevado-Holgado AJ, Kormilitzin A, Shi L, Hye A, Ashton NJ, Morgan AR, Bos I, Vos SJB, Baker S, Buckley NJ, Ten Kate M, Scheltens P, Teunissen CE, Vandenberghe R, Gabel S, Meersmans K, Engelborghs S, De Roeck EE, Sleegers K, Frisoni GB, Blin O, Richardson JC, Bordet R, Molinuevo JL, Rami L, Wallin A, Kettunen P, Tsolaki M, Verhey F, Lléo A, Sala I, Popp J, Peyratout G, Martinez-Lage P, Tainta M, Johannsen P, Freund-Levi Y, Frölich L, Dobricic V, Legido-Quigley C, Bertram L, Barkhof F, Zetterberg H, Morgan BP, Streffer J, Visser PJ, Lovestone S. (2020). Validation of Plasma Proteomic Biomarkers Relating to Brain Amyloid Burden in the EMIF-Alzheimer's Disease Multimodal Biomarker Discovery Cohort. J Alzheimers Dis. 74(1):213-225. doi: 10.3233/JAD-190434. PMID: 31985466.

## <u>Reviewer 3</u>:

**Comment 1:** The paper by Prasad et al address an important unmet need for better patient stratification in the clinical management of arthritis. The treatments for arthritis are expensive, not universally effective and are currently administered on a trial and error basis meaning control of symptoms is sub-optimal for many patients. The study uses circulating proteins to predict response to TNFi after six months of care and identified a number of proteins that may be useful in stratification.

**Response 1:** Authors would like to thank the reviewer for pointing out the unmet need for such a predictor in clinical setting and relevance of our predictor.

**Comment 2:** I have some general and specific comments. General: It is my understanding that the raw data and the analysis methods (e.g. scripts) should be made available in an appropriate online repository. The authors state that this is achieved via a GitHub page but it is not clear to me that the raw data and analysis scripts used to perform the analysis as presented are available in this location. If I understand correctly, this is a prerequisite for publication in PLOS Computational Biology and should be addressed.

**Response 2:**

We have now included all the scripts used in the analysis methods in the following folder of our Github repository: https://github.com/ShuklaLab/ATRPred/tree/main/scripts

We have now included the raw data in the following folder of our Github repository: https://github.com/ShuklaLab/ATRPred/tree/main/raw_data

Please also see lines 595-597 in the "changes highlighted" version of the manuscript, which reflect the above mentioned updates.

**Comment 3:** The paper could be condensed in several sections to. For example, the introduction could be shortened and as well as the discussion (particularly between lines 91 to 104 and 455 to 472).

**Response 3:** We have now consolidated the introduction, methods, results, and discussion sections. Please see highlighted lines 98-101, 194-196, 267-270, 325-330, and 477-492 in the "changes highlighted" version of the manuscript.

**Comment 4:** In the abstract the authors state "We then labelled these patients as responders (n=60) and non-responders (n=29) based on the baseline disease activity score (BLDAS)." This seems a bit misleading as EULAR criteria applied at 6-months was used?

**Response 4:** Authors confirm that the anti-TNF response of patients was obtained at 6 months following BSR criteria. We have now edited the above mentioned line of our abstract to reflect this correction. Please see highlighted changes in line numbers 35-36 of the "changes highlighted" version of the manuscript.

**Comment 5:** Specific: The authors state that of 144 samples sent to Olink 1/3 (n=55) failed QC – Can the authors explain why this failure rate was so high?

**Response 5:** A large number of patients (144 – 89 = 55) were lost to follow-up; either were not able to make 6 months follow-up appointment or couldn't recover, and every composite require to calculate DAS score was not available. Follow-up DAS score was required for knowing a patient's response to anti-TNF and for modelling the ML predictor. Also, not all 144 patients were on anti-TNF; some of them were on other biologic DMARDs (such as Tociluzimab, Ritiuximab, etc). That is why the sample size got reduced for Olink proteomics data analysis. For all the 89 patients' samples for which we had follow-up DAS score, we can confirm that there was not any quality degradation in the protein quantification. So, there was no QC failure for any samples. We have now edited the manuscript to reflect this information. Please see highlighted lines 170-173 in the "changes highlighted" version of the manuscript.

**Comment 6:** The biological Importance of PCA based grouping of patients is very unclear and adds little to the paper. Could the author possibly add more detail here e.g. identify which proteins account for the perceived separation?

**Response 6:** The separation seen in PC3 explains only 5.4% variance of the whole Olink proteomics dataset (new Supplementary Figure 1A). We have now also included the variance captured in the Figure 1. Since this separation by PC3 was based on all 352 proteins which were able to explain only 5.4% of variance, we didn't pursue the proteins accounting for this separation. However, the separation made by PC3 was striking, and therefore we further pursued the statistical analysis of clinical features driving those two clusters and found that baseline DAS and gender were in fact significant (Table 2). Literature review indeed suggests these two features are confounders of protein expression; thus, reinforcing our biological understanding. We agree with the reviewer that the 2 classes (endotypes) seen do not provide much interest as they are mainly based on two confounders (gender and BLDAS), as also already discussed in our paper. However, we decided to keep this figure 1 in the paper, as it reinforces our biological understanding of the data.

In the discussion section of the "changes highlighted" version of our manuscript, we have earlier given following explanation for the relevance of this part of the study:

Line numbers 460-472:

*"The current study uncovered two distinct endotypes based on the expression profile of all 352 plasma proteins, which had significantly different gender proportions and baseline DAS (Figure 1 and Table 2). Since, these endotypes were not significantly different in terms of their anti-TNF treatment response (Table 2), there is a possibility of the existence of two distinct RA disease endotypes, which may be important in other aspects of the disease management or other drug response. Gender is known to be significantly associated with plasma protein profile (Silliman et al., 2012). Further, DAS28 is also known to be correlated with the plasma proteins such as IL37 (Xia et al., 2015) and CXCL10 (Pandya et al., 2017). A significantly higher average ESR has been observed in females of age up to 75 years (Wetteland et al., 1996). Considering the above literature, there is another possibility that the two endotypes uncovered in this study may be totally unrelated to RA. Hence, the clinicians may consider keeping a strict vigil on these endotypes, which may be helpful in better informed decision making."*

Line numbers 575-580:

*"We have identified two clusters (Figure 1 and Table 2) driven by plasma protein profile as a plausible endotypes. Unfortunately, they do not correspond to anti-TNF therapy responsiveness, but they are still significantly different in terms of disease activity and gender, and thus possibly play an important role in patient management. For example, since these endotypes are independent of future treatment response, they may indicate pre-biologic treatment pathology sub-groups, which can be investigated in future studies."*

**Comment 7:** Could the author give more information on the TNFi therapy - was a specific TNFi used or were all TNFi permitted? Did the TNFi type differ between the responder groups? Were the patients exposed to TNFi for the first time? This information should be added to the table of patient characteristics.

**Response 7:** There were multiple anti-TNFs (*viz.* adalimumab, etanercept, infliximab, certolizumab or golimumab). We have now included this information in patient characteristics (updated Table 1). Adalimumab was the most popular drug prescribed. Since numbers after splitting the samples based on different anti-TNFs are small and adalimumab was the most popular prescribed drug (updated Table 1), statistics on comparing different anti-TNFs between the two groups was not useful. Moreover, our aim was to create a general anti-TNF predictor irrespective of which anti-TNF drug is prescribed.

Yes, patients were exposed to anti-TNF for the first time. Please see line 140 of the "changes highlighted" version of the manuscript, which says *"… naïve to biologic DMARDs …"*.

**Comment 8:** My main concern is the limited sample size and the lack of true independent validation. The entire cohort included only 29 non-responders. Once you get down to the inner folds of the nested CV these numbers are starting to get extremely small. Although the authors

took care to design the experiment to minimise bias due to only having a single dataset, I'm still concerned that all samples were touched by the statistical approach and no truly independent samples were available for validation. A completely separate validation sample would strengthen the paper considerably.

**Response 8:** Authors would like to thank reviewer to mention that we have tried our best in minimizing the bias as far as possible for the small dataset. We have used a nested cross-validation scheme, so that the outer cross-validation provides an estimate of the performance, which is not biased by selection of test data. Authors agree with the reviewer for the need of a validation study on a completely independent cohort. Patient recruitment is an expensive and tedious process. However, going forward, we will do a validation study to re-confirm the performance of ATRPred on a completely independent cohort. We plan to apply for research grants based on this current paper to progress our research further. Many papers have published an even smaller sample size for the discovery cohort as compared to ours (N=89). For example, Westwood et al. (2016) reported plasma biomarkers for their discovery cohort (N=79) and later they validated their results in a follow-up study (Westwood et al., 2020).

References:


1. Westwood S, Leoni E, Hye A, Lynham S, Khondoker MR, Ashton NJ, Kiddle SJ, Baird AL, Sainz-Fuertes R, Leung R, Graf J, Hehir CT, Baker D, Cereda C, Bazenet C, Ward M, Thambisetty M, Lovestone S. (2016). Blood-Based Biomarker Candidates of Cerebral Amyloid Using PiB PET in Non-Demented Elderly. J Alzheimers Dis. 52(2):561-72. doi: 10.3233/JAD-151155. PMID: 27031486.
2. Westwood S, Baird AL, Anand SN, Nevado-Holgado AJ, Kormilitzin A, Shi L, Hye A, Ashton NJ, Morgan AR, Bos I, Vos SJB, Baker S, Buckley NJ, Ten Kate M, Scheltens P, Teunissen CE, Vandenberghe R, Gabel S, Meersmans K, Engelborghs S, De Roeck EE, Sleegers K, Frisoni GB, Blin O, Richardson JC, Bordet R, Molinuevo JL, Rami L, Wallin A, Kettunen P, Tsolaki M, Verhey F, Lléo A, Sala I, Popp J, Peyratout G, Martinez-Lage P, Tainta M, Johannsen P, Freund-Levi Y, Frölich L, Dobricic V, Legido-Quigley C, Bertram L, Barkhof F, Zetterberg H, Morgan BP, Streffer J, Visser PJ, Lovestone S. (2020). Validation of Plasma Proteomic Biomarkers Relating to Brain Amyloid Burden in the EMIF-Alzheimer's Disease Multimodal Biomarker Discovery Cohort. J Alzheimers Dis. 74(1):213-225. doi: 10.3233/JAD-190434. PMID: 31985466.


**Comment 9:** Due to the imbalanced nature of the outcome, did the authors consider oversampling the non-responders and if not why was this decided not to be important?

**Response 9:** Authors agree the data imbalance can cause a potential bias for the predictor towards the majority class. We did not do oversampling of non-responders, as this created scepticism in the co-author clinicians we worked with. However, to address this potential bias, we included a performance metric Matthew's Correlation Coefficient (MCC), which is a good

metric in capturing performance even in imbalance sets (Chicco and Jurman, 2020). The value of MCC varies from -1 to 1 and we were able to achieve a value of 0.6 as a performance for our final ML predictor.

Reference:

1. Chicco, D., Jurman, G. (2020). The advantages of the Matthews correlation coefficient (MCC) over F1 score and accuracy in binary classification evaluation. BMC Genomics 21, 6. doi: 10.1186/s12864-019-6413-7.

**Comment 10:** Table 1 and Figure 1. It looks like there are some quite low baseline DAS scores if I read correctly? However, I think this cannot be the case as a DAS score >5.1 at pre-treatment is an inclusion criteria?

**Response 10:** Authors agree that DAS score >5.1 was the inclusion criteria at the biologic (anti-TNF) assessment time, which preceded the baseline of the study. However, for some of the patients, it dipped a little on the day when the first dose of anti-TNF treatment was given. We chose to use this value for baseline DAS (BLDAS) as it was the day when the anti-TNF treatment was first administered to this biologic naïve cohort.

**Comment 11:** ML methods are repeated in the results section. Please consolidate in the methods and remove from results.

**Response 11:** We have now consolidated the results. Please see highlighted lines 267-270 and 321-326 in the “changes highlighted” version of the manuscript.
